# Mobile Health App Acceptance in Japan’s Aging Society: Multigroup Structural Equation Modeling Based on the Extended Unified Theory of Acceptance and Use of Technology and eHealth Literacy Frameworks

**DOI:** 10.2196/87832

**Published:** 2026-06-09

**Authors:** Yasue Fukuda, Koji Fukuda

**Affiliations:** 1 Faculty of Pharmaceutical Sciences Suzuka University of Medical Science Suzuka, Mie Japan; 2 Faculty of Political Science and Economics Waseda University Tokyo, Shinjuku-ku Japan

**Keywords:** aging society, distrust, eHealth literacy, health interest, Japan, mHealth, mobile health, self-efficacy, structural equation modeling, Unified Theory of Acceptance and Use of Technology, UTAUT

## Abstract

**Background:**

Mobile health (mHealth) technologies are increasingly promoted as tools for chronic disease management and healthy aging, yet adoption remains persistently uneven across demographic groups. Japan, where 29.1% of the population is 65 years or older—the highest proportion globally—exemplifies the challenges of mHealth promotion in super-aging societies. Despite high smartphone penetration (90.1%) and active national digital transformation initiatives, only 21.6% of Japanese adults report regular mHealth app use, with marked disparities by age and sex.

**Objective:**

This study examined determinants of mHealth acceptance by extending the unified theory of acceptance and use of technology to incorporate eHealth literacy, self-efficacy, perceived risk, distrust, and health-related factors (health status and health interest). Age- and sex-specific differences in acceptance mechanisms were also investigated using multigroup structural equation modeling (SEM).

**Methods:**

We conducted a cross-sectional online survey in November 2023 with 960 Japanese adults sampled across 7 age strata (aged 18-27 years to aged ≥78 years). SEM tested hypothesized relationships among 9 constructs. Health status and health interest were included as observed covariates. Multigroup SEM with configural, metric, and structural invariance testing examined age- and sex-specific differences, and binary logistic regression identified predictors of current mHealth app use.

**Results:**

The structural model demonstrated good fit (*χ*^2^/*df*=2.06; comparative fit index 0.953; Tucker-Lewis index 0.945; root mean square error of approximation 0.047) and explained 71.6% of the variance in behavioral intention. Effort expectancy (*β*=0.404), facilitating conditions (*β*=0.349), and performance expectancy (*β*=0.188) were the primary proximal predictors of behavioral intention. Social influence exerted strong upstream effects on effort expectancy (*β*=0.811), eHealth literacy (*β*=0.507), and self-efficacy (*β*=0.422). Health interest positively influenced performance expectancy (*β*=0.133), whereas neither health interest nor health status showed a significant direct effect on distrust. Distrust did not directly predict behavioral intention in the overall sample. Multigroup analyses identified 5 significant age differences and 5 sex differences. eHealth literacy increased distrust among young adults but reduced perceived risk among middle-aged and older adults. Self-efficacy negatively predicted performance expectancy among young adults yet positively predicted it among middle-aged and older adults. Distrust significantly reduced behavioral intention only among middle-aged adults.

**Conclusions:**

mHealth acceptance in Japan’s aging society is characterized by stable proximal determinants of behavioral intention alongside heterogeneous upstream belief formation processes that vary systematically by age and sex. Health interest, rather than health status, emerged as the key contextual driver of perceived usefulness. At the theoretical level, this study clarifies how eHealth literacy, self-efficacy, and distrust function as age- and sex-contingent antecedents within an extended unified theory of acceptance and use of technology framework. At the practical level, these findings highlight the need for trust-centered, demographically tailored, and literacy-sensitive strategies to promote equitable mHealth adoption in rapidly aging societies.

## Introduction

### Background

Mobile health (mHealth) technologies have emerged as critical tools for health care delivery, particularly in aging societies facing resource constraints and increasing chronic disease burdens [1,2]. Japan, with 29.1% of its population aged ≥65 years and projected to reach 38.4% by 2065 [3], exemplifies these demographic challenges [4]. Despite widespread smartphone penetration of 90.1% in 2022 and advanced digital infrastructure [5], mHealth adoption remains paradoxically low, with only 21.6% of Japanese adults reporting regular use of health-related apps [6,7]. This paradox reveals fundamental gaps in understanding technology acceptance mechanisms, particularly in aging populations where mHealth could deliver the greatest health benefits [8,9].

Existing technology acceptance research has predominantly examined younger, technologically proficient users in Western contexts [10,11], creating a critical knowledge gap regarding older adults’ adoption mechanisms in rapidly aging Asian societies. Moreover, traditional technology acceptance models often overlook psychosocial factors specific to health technologies, privacy concerns [12,13], system distrust [14,15], and eHealth literacy [16,17], which may operate differently across age cohorts and cultural contexts. Understanding these age-differentiated, context-specific acceptance mechanisms is essential for designing evidence-based interventions to promote mHealth adoption among the world’s fastest-aging populations [18,19].

### Research Gaps and Theoretical Contributions

Despite extensive unified theory of acceptance and use of technology (UTAUT) applications to diverse technologies [20-22], several critical limitations persist in mHealth adoption research. Most existing studies have examined general populations or younger cohorts [23,24], with insufficient attention to older adults’ distinct needs, capabilities, and concerns in aging societies where they constitute the primary target population for disease management apps yet demonstrate the lowest adoption rates [25,26]. In Japan’s context, prior mHealth acceptance research has primarily focused on specific demographic segments to enable in-depth investigation. For example, Cao et al [27] examined mHealth acceptance among Japanese young adults using an extended UTAUT model, finding that trust, performance expectancy, and effort expectancy directly influenced behavioral intention, while health consciousness and social influence operated indirectly. Building upon these valuable insights, a comprehensive examination across the full adult lifespan within a single study remains needed to identify life stage–specific intervention strategies and enable population-level policy formulation [28,29].

Furthermore, research rarely examines how acceptance mechanisms vary across demographic subgroups within the same population [30,31]. While some studies compare age or sex groups separately, few use rigorous multigroup structural equation modeling (SEM) to test whether theorized relationships operate identically across subgroups [32,33], preventing identification of demographic-specific barriers and facilitators [34,35]. Additionally, few studies distinguish between disease-management apps and wellness-oriented apps, despite potentially distinct adoption drivers [36,37].

This study complements existing research through 3 theoretical contributions. First, we integrate privacy-trust dimensions by incorporating perceived risk [25,38] and distrust [30,39] as distinct constructs—dimensions inadequately addressed in core UTAUT yet critical in health contexts involving sensitive personal data [40,41]. Importantly, we theorize distrust not merely as a direct barrier to behavioral intention but as a mediating mechanism undermining other acceptance perceptions, proposing that skepticism operates through both direct and indirect pathways [42,43]. This dual-pathway conceptualization advances prior research treating distrust solely as a direct predictor [14,30].

Second, we theorize eHealth literacy [16,17] as a multifaceted construct that simultaneously enhances acceptance perceptions while potentially increasing critical risk evaluation. This dual-function conceptualization challenges prevailing assumptions that digital literacy uniformly reduces barriers [44,45]. We hypothesize that eHealth literacy may paradoxically increase distrust among digitally sophisticated users capable of critical evaluation—an alternative mechanism unexplored in prior research [46,47].

Third, we extend UTAUT by theorizing social influence as a comprehensive distal predictor affecting not only core UTAUT constructs but also self-efficacy, eHealth literacy, and distrust [20,48,49]. This comprehensive pathway specification tests whether social influence operates through multiple mechanisms simultaneously—a theoretical proposition inadequately examined in prior research [20,48].

### Theoretical Model and Research Hypotheses

#### Conceptual Framework

**Figure 1:**
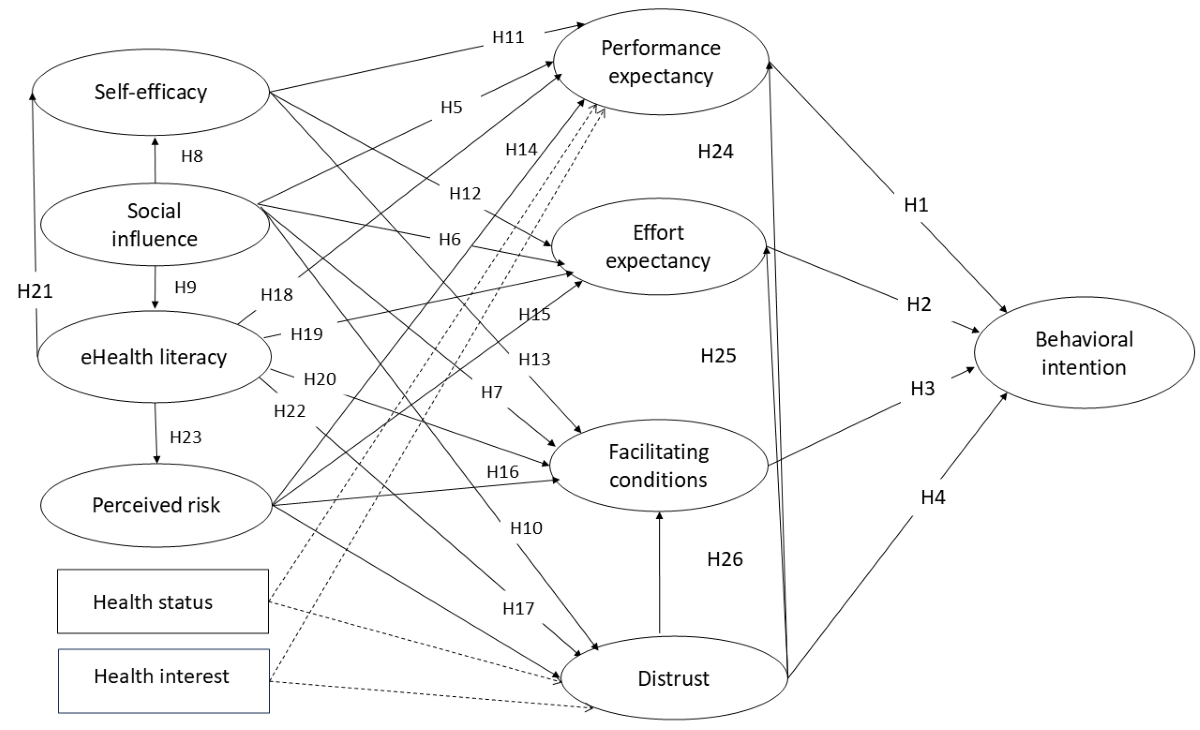
Extended unified theory of acceptance and use of technology research model incorporating eHealth literacy, self-efficacy, perceived risk, distrust, and health-related covariates (H1-H26). Dotted lines indicate covariate effects.

The theoretical model integrates core UTAUT constructs with privacy-trust dimensions and digital literacy constructs to comprehensively explain mHealth acceptance mechanisms [20,50]. The model comprises 8 constructs organized into 3 layers: social influence, self-efficacy, perceived risk, and eHealth literacy function as distal predictors; performance expectancy, effort expectancy, facilitating conditions, and distrust serve as proximal predictors; and behavioral intention represents the outcome construct [51,52]. This multilayered specification enables examination of direct effects, indirect effects through mediation pathways, and total effects across demographic subgroups and app types [53,54]. Figure 1 presents the theoretical model with all hypothesized relationships.

#### Research Hypotheses

Based on UTAUT [20,50] and theoretical extensions [17,25,30], we propose 26 hypotheses specifying relationships among constructs [55,56].

- H1: performance expectancy positively influences behavioral intention to use mHealth apps [20,57]. Users perceiving greater health benefits demonstrate higher adoption intentions.
- H2: effort expectancy positively influences behavioral intention to use mHealth apps [20,57]. Users perceiving ease of use demonstrate higher adoption intentions.
- H3: facilitating conditions positively influence behavioral intention to use mHealth apps [20,57]. Users perceiving adequate infrastructure support demonstrate higher adoption intentions.
- H4: distrust negatively influences behavioral intention to use mHealth apps [30,58,59]. System skepticism directly discourages adoption.
- H5: social influence positively influences performance expectancy [20,48]. Social endorsements enhance perceived benefits through observational learning.
- H6: social influence positively influences effort expectancy [20,48]. Peer success experiences enhance ease-of-use perceptions.
- H7: social influence positively influences facilitating conditions [20,48]. Social networks provide information about support resources.
- H8: social influence positively influences self-efficacy [48,60]. Observing others’ success enhances confidence through vicarious experience.
- H9: social influence positively influences eHealth literacy [48,49]. Social environments facilitate learning through knowledge exchange.
- H10: social influence negatively influences distrust [49]. Trusted endorsements reduce system skepticism through trust transfer.
- H11: self-efficacy positively influences performance expectancy [60,61]. Confident users anticipate successfully extracting maximum app value.
- H12: self-efficacy positively influences effort expectancy [60,61]. Confident users perceive technology tasks as less effortful.
- H13: self-efficacy positively influences facilitating conditions [60]. Confident users perceive support infrastructure as more adequate.
- H14: perceived risk negatively influences performance expectancy [25,38]. Privacy concerns introduce costs that offset anticipated benefits.
- H15: perceived risk negatively influences effort expectancy [25,38]. Privacy concerns increase perceived complexity through risk management burden.
- H16: perceived risk negatively influences facilitating conditions [38,62]. Privacy concerns signal potential infrastructure inadequacies.
- H17: perceived risk positively influences distrust [62,63]. Privacy concerns amplify general system skepticism.
- H18: eHealth literacy positively influences performance expectancy [16,17]. Digital health competencies enable informed benefit recognition.
- H19: eHealth literacy positively influences effort expectancy [16,17]. Digital literacy reduces learning curves through transferable skills.
- H20: eHealth literacy positively influences facilitating conditions [17,64]. Digital competencies expand perceived scope of adequate support.
- H21: eHealth literacy positively influences self-efficacy [16,64]. Digital health competencies enhance confidence through mastery experiences.
- H22: eHealth literacy negatively influences distrust [46,47]. Digital understanding may reduce skepticism, though sophisticated users may paradoxically identify system vulnerabilities.
- H23: eHealth literacy negatively influences perceived risk [44,45]. Digital knowledge enables accurate risk assessment that typically reduces threat overestimation.
- H24: distrust negatively influences performance expectancy [65,66]. Skepticism introduces doubts about whether promised benefits will materialize.
- H25: distrust negatively influences effort expectancy [65,66]. Skepticism elevates perceived complexity through suspicion of deliberately confusing design.
- H26: distrust negatively influences facilitating conditions [66,67]. Skepticism reduces confidence that adequate support truly exists.

Together, H24-H26 specify distrust’s indirect pathways complementing the direct effect in H4, capturing how system skepticism operates through both immediate discouragement and systematic erosion of acceptance perceptions.

In addition, self-rated health status and health interest were incorporated as observed exogenous covariates predicting performance expectancy and distrust. This specification allows examination of whether perceived health condition and baseline health motivation shape core acceptance beliefs, thereby addressing the role of health-related need in mHealth adoption.

### Research Objectives

This study pursued 3 objectives. First, we tested an extended UTAUT model incorporating perceived risk, distrust, eHealth literacy, and health-related covariates (health status and health interest), evaluating all 26 hypothesized relationships.

Second, we examined demographic heterogeneity by testing whether structural relationships differ across age groups (young adults aged 18-39 years, middle-aged adults aged 40-59 years, and older adults aged ≥60 years) and sex groups using multigroup SEM.

Third, we explored app-type-specific adoption mechanisms for different mHealth apps.

Hypotheses were specified for the overall model, whereas subgroup and app-type analyses were conducted as comparative extensions.

## Methods

### Ethical Considerations

This cross-sectional online survey study examined determinants of mHealth adoption in Japan’s aging society between November 11 and 18, 2023. Ethical approval was obtained from the Waseda University Ethics Committee (number 2023-250), and the study complied with Japan’s Act on the Protection of Personal Information [41]. Electronic informed consent was obtained from all participants through the online survey platform. Participants received a small number of reward points corresponding to approximately ¥10 or less than US $0.10 for completing the survey. No personally identifiable information was collected, and study reporting followed the CHERRIES (Checklist for Reporting Results of Internet E-Surveys; Multimedia Appendix 1) guidelines [68]. Survey data were stored on secure, password-protected servers accessible only to the research team and were exported to encrypted files for analysis. All analyses used deidentified data, and results are reported only in aggregate so that no individual participant can be identified.

### Participants and Sampling

Sample size was determined using 2 approaches. For population estimation, normal approximation with finite-population correction for Japan (126 million) required a minimum n=385 (95% confidence; 5% margin of error; *P*=.50). For SEM (9 constructs; 31 indicators; 26 paths), a minimum n=310 was required (10:1 observations-to-parameters ratio) [69,70]. For multigroup analyses across 3 age groups and 2 sexes, 100 to 150 participants per subgroup ensured adequate power [32,33]. The target sample (N=960) exceeded all requirements and provided power >0.80 for detecting moderate effects (*β*≥0.20). Age was collected as a continuous variable (in years) and was also categorized into seven 10-year age strata for quota sampling and descriptive purposes.

Participants were recruited from the Cross Marketing, Inc (Tokyo, Japan) internet research panel using quota sampling across 7 age strata (18-27 years, 28-37 years, 38-47 years, 48-57 years, 58-67 years, 68-77 years, and ≥78 years) to ensure representation across the adult lifespan.

Of 23,434 invited panelists, a total of 2203 consented to participate. Data quality was ensured through multiple procedures, including the exclusion of responses with inconsistent demographic information and extremely short completion times. In addition, an attention-check item was embedded within the 5-point Likert scale questionnaire (eg, “Please select ‘strongly agree’ for this item”). Respondents who selected any option other than the instructed response were considered inattentive and excluded from the final sample.

After applying these criteria, a total of 960 valid responses were retained for analysis.

For inferential analyses, these 7 strata were collapsed into 3 theoretically meaningful groups: young adults (aged 18-39 years), middle-aged adults (aged 40-59 years), and older adults (aged ≥60 years), ensuring adequate statistical power for multigroup SEM. We chose 3 life-stage groups rather than all seven 10-year strata to avoid very small subgroup sizes, which would have produced unstable parameter estimates and less interpretable age-specific patterns in the SEM.

Participants were not required to have prior experience with mHealth apps, as the study assessed both behavioral intention and current use. However, all participants necessarily had internet access and basic digital literacy due to the online survey format.

### Measurement Instruments

Participants reported demographic variables (age, sex, marital status, educational level, and current use of health monitoring apps). Technology acceptance constructs were measured using 9 established scales, each via 5-point Likert items (1=strongly disagree and 5=strongly agree). The following latent constructs were assessed: performance expectancy, effort expectancy, social influence, facilitating conditions, privacy risk, self-efficacy, distrust, behavioral intention, and eHealth literacy (eHealth Literacy Scale).

All survey items and their sources are provided in Table S1 in Multimedia Appendix 2. Items served as observed variables for latent constructs in SEM. Measurement validity and reliability were evaluated using confirmatory factor analysis (CFA).

### Statistical Analysis

All analyses were performed using IBM SPSS Statistics (version 30) and IBM SPSS AMOS (version 30) [71]. Statistical significance was set at *P*<.05. Descriptive statistics summarized participant characteristics. Internal consistency was assessed using Cronbach α [72]. Multicollinearity was assessed using the variance inflation factor <5 [71,72].

CFA tested measurement validity, establishing adequacy via standardized factor loadings [72,73], average variance extracted (AVE≥0.50), and composite reliability (CR≥0.70). Discriminant validity was determined using the Fornell-Larcker criterion [72].

SEM using maximum likelihood estimation was performed to test hypothesized paths [71,74]. In the structural model, self-rated health status and health interest were specified as observed covariates predicting performance expectancy and distrust to account for individual differences in perceived health condition and baseline health motivation. Model fit was evaluated using multiple indices: *χ*^2^/df<3, CFI>.90, TLI>.90, goodness-of-fit index (GFI)>.90, adjusted GFI (AGFI)>.90, and root mean square error of approximation (RMSEA)≤.08 [72,73,75,76]. Descriptive statistics and inter-construct correlations appear in Table S2 in Multimedia Appendix 3.

Multi-group SEM examined sex and age differences with configural, metric, and structural invariance testing [32,33]. Chi-square difference tests (Δ*χ*^2^) evaluated group differences [77,78]. Binary logistic regression analysis identified predictors of mHealth app use and behavioral intention by app type, reporting odds ratios (ORs) and 95% CIs [71,72]. Binary logistic regression analysis was used for app-specific use patterns because the outcome variable was dichotomous (current use vs nonuse) and the number of current users per app type was insufficient for stable SEM estimation (blood pressure: n=173; heart rate: n=118; physical activity: n=110). For the app-specific logistic regression analyses, construct scores were calculated as the mean scores of the corresponding questionnaire items and used as independent variables.

## Results

### Participant Characteristics

Among 960 valid respondents (476/960, 49.6% male; 484/960, 50.4% female; mean age 51.8, SD 17.8 years), participants were stratified into young adults (aged 18-39 years; 298/960, 31%), middle-aged adults (aged 40-59 years; 275/960, 28.6%), and older adults (aged ≥60 years; 387/960, 40.3%). For descriptive purposes, Table 1 reports age distributions across the original seven 10-year strata used for quota sampling, whereas subsequent inferential analyses (multigroup SEM and logistic regression) are based on the 3 life-stage groups (young adults, middle-aged adults, and older adults). Educational attainment was high, with university or graduate degrees representing the largest category (482/960, 50.2%), followed by high school graduates (272/960, 28.3%) and junior college graduates (156/960, 16.3%). Most participants were married (633/960, 65.9%), reported health interest (786/960, 81.9%), and self-assessed their health status as good or somewhat better (526/960, 54.8%).

**Table 1:** Demographic characteristics of participants (N=960).

Demographic characteristics		Frequency, n (%)
Age (years)		
	18-27	130 (13.5)
	28-37	138 (14.4)
	38-47	138 (14.4)
	48-57	143 (14.9)
	58-67	136 (14.2)
	68-77	138 (14.4)
	≥78	137 (14.3)
Sex		
	Male	476 (49.6)
	Female	484 (50.4)
Education		
	Junior high school graduate	31 (3.2)
	High school graduate	272 (28.3)
	Junior college or vocational school graduate	156 (16.3)
	University graduate or master’s-level graduate	482 (50.2)
	I do not want to answer	19 (2)
Marital status		
	Unmarried	318 (33.1)
	Married	633 (65.9)
	I do not want to answer	9 (0.9)
Interest in health		
	Not interested at all	22 (2.3)
	Not very interested	40 (4.2)
	Neither one nor the other	104 (10.8)
	Somewhat curious	387 (40.3)
	Very interested	399 (41.6)
	Do not know	8 (0.8)
Health status		
	Bad	26 (2.7)
	Somewhat worse	153 (15.9)
	Neither one nor the other	252 (26.3)
	Somewhat better	364 (37.9)
	Good	162 (16.9)
	I do not want to answer	3 (0.3)

### eHealth Literacy

Table 2 provides the results on eHealth literacy by sex, age, and educational background.

The overall mean eHealth literacy score was 22.84 (SD 6.31). No significant sex differences were observed (male: 22.90, SD 6.72 and female: 22.79, SD 5.98), nor were there significant differences by educational level. However, age-related differences were identified, with participants aged 28-37 years (mean 24.42, SD 6.22) demonstrating the highest eHealth literacy scores, while those aged ≥78 years showed the lowest scores (mean 20.98, SD 6.14).

**Table 2:** eHealth literacy and demographic characteristics of participants.

Demographic characteristics and eHealth literacy		Mean (SD)
Age (years)		
	18-27	23.1077 (7.20922)
	28-37	24.4203 (6.22429)
	38-47	23.6739 (6.00871)
	48-57	22.7063 (6.58298)
	58-67	22.5515 (5.53082)
	68-77	22.4493 (6.28644)
	≥78	20.9781 (6.14347)
Sex		
	Male	22.8971 (6.72309)
	Female	22.7851 (5.98059)
Education		
	Junior high school graduate	20.7097 (7.52415)
	High school graduate	22.4044 (5.85354)
	Junior college or vocational school graduate	22.859 (6.63271)
	University graduate or master’s-level graduate	23.3631 (6.39248)
	I do not want to answer	19.1579 (6.27396)
	Total	22.8406 (6.3053)

### Descriptive Statistics for Study Constructs

Descriptive statistics (mean and SD) for all constructs and questionnaire items are provided in Table S2 in Multimedia Appendix 3. Across constructs, mean values for behavioral intention, distrust, and performance expectancy tended to be higher than for perceived risk and social influence, indicating broadly positive expectations for mHealth alongside some skepticism toward information reliability.

### Current Use of mHealth Apps

Regarding mHealth use, a substantial majority (688/960, 71.7%) reported no current use, while 272/960 (28.3%) used at least 1 app. Among those, blood pressure monitoring showed the highest usage (173/960, 18.0%), followed by heart rate monitoring (118/960, 12.3%) and physical activity tracking (110/960, 11.5%). Detailed usage rates for each type of mHealth app are presented in Multimedia Appendix 4.

Usage patterns differed significantly by age group, with older adults demonstrating higher adoption of disease-management apps, while younger adults showed greater engagement with wellness-oriented apps.

### Measurement Model Validation

Table 3 summarizes the convergent and discriminant validity indices (standardized loading ranges, AVE, CR, Cronbach α, maximum shared variance, and discriminant validity judgments) for all constructs included in the measurement model. The measurement model demonstrated satisfactory psychometric properties, with all standardized factor loadings exceeding 0.60 and most exceeding 0.70 (Table S3 in Multimedia Appendix 5 and Table S2 in Multimedia Appendix 3).

Convergent validity was supported, as most constructs achieved AVE values above the recommended threshold of 0.50. Self-efficacy and distrust were at the threshold level (approximately 0.50) but remained acceptable given their CR>0.70 and substantial factor loadings. To further address the marginal AVE values for self-efficacy and distrust, we estimated alternative CFA and structural models in which the lowest-loading indicator of each construct was removed. These reduced-item models yielded slightly higher AVE estimates and marginally improved fit indices (eg, CFI≈0.95; RMSEA≈0.05), but the overall pattern and significance of structural paths remained essentially unchanged. We therefore retained the original 3-item scales for self-efficacy and distrust in the main model to preserve content validity and comparability with prior research, while interpreting their structural effects with appropriate caution.

Discriminant validity was confirmed using the Fornell-Larcker criterion, as all constructs demonstrated AVE values greater than their maximum shared variance.

**Table 3:** Measurement model indices and discriminant validity (N=960). Discriminant validity was confirmed via the Fornell-Larcker criterion (average variance extracted [AVE] > maximum shared variance [MSV]). Detailed factor loadings are available in Table S2 in Multimedia Appendix 3. AVE values for self-efficacy and distrust were near the 0.50 threshold but were considered acceptable given composite reliability (CR) >0.70 and substantial factor loadings.

Construct	Standardized loading range	AVE	CR	Cronbach α	MSV	Discriminant validity
Self-efficacy	0.656-0.823	0.501	0.752	0.731	0.214	Supported
Social influence	0.767-0.824	0.644	0.844	0.871	0.54	Supported
Privacy risk	0.659-0.921	0.658	0.852	0.83	0.035	Supported
Performance expectancy	0.788-0.860	0.687	0.868	0.868	0.578	Supported
Effort expectancy	0.799-0.825	0.659	0.795	0.779	0.578	Supported
Distrust	0.612-0.773	0.5	0.749	0.739	0.035	Supported
Facilitating conditions	0.693-0.848	0.65	0.847	0.827	0.578	Supported
Behavioral intention	0.841-0.901	0.761	0.905	0.907	0.543	Supported
eHealth literacy	0.745-0.872	0.665	0.941	0.935	0.367	Supported

### Overall Structural Model

The hypothesized extended UTAUT model is shown in Figure 1. The extended structural equation model including health interest and health status demonstrated good overall fit to the data: *χ*^2^_452_=932.4; *P*<.001; *χ*²/df=2.1; GFI=0.889; AGFI=0.863; CFI=0.953; TLI=0.945; RMSEA=0.047 (90% CI 0.043-0.052). Although the chi-square statistic was statistically significant, which is common in large samples, the ratio of chi-square to degrees of freedom remained within an acceptable range. In addition, both incremental and absolute fit indices met or exceeded commonly recommended thresholds, indicating adequate model fit.

The structural model explained a substantial proportion of variance in behavioral intention (*R*^2^=0.716). The explanatory power for endogenous constructs varied across variables, ranging from 0.4% for perceived risk to 82.1% for effort expectancy. Facilitating conditions (*R*^2^=0.666) and performance expectancy (*R*^2^=0.628) also showed substantial explained variance. These results indicate that the extended UTAUT framework provides strong explanatory power for mHealth acceptance in the study sample.

The structural path coefficients for the overall sample are presented in Table 4.

Effort expectancy exerted the strongest positive effect on behavioral intention (*β*=0.404; *P*<.001), followed by facilitating conditions (*β*=0.349; *P*<.001) and performance expectancy (*β*=0.188; *P*<.001), supporting H1-H3. In contrast, distrust did not have a statistically significant effect on behavioral intention (*β*=−0.027; *P*=.45); therefore, H4 was not supported. Together, these findings indicate that perceived ease of use, infrastructural support, and expected health benefits are the primary proximal determinants of mHealth adoption intention in the overall sample, while distrust does not exert a direct influence.

Social influence emerged as the most powerful distal predictor in the model. It showed strong positive effects on effort expectancy (*β*=0.811; *P*<.001), facilitating conditions (*β*=0.548; *P*<.001), and performance expectancy (*β*=0.662; *P*<.001), supporting H5-H7. Social influence also significantly enhanced self-efficacy (*β*=0.422; *P*<.001) and eHealth literacy (*β*=0.507; *P*<.001), supporting H8 and H9. In addition, social influence significantly reduced distrust (*β*=−0.248; *P*<.001), supporting H10. These results indicate that social environments strongly shape perceptions of usefulness, ease of use, infrastructural support, digital competencies, and trust in mHealth technologies.

eHealth literacy demonstrated a complex pattern of effects. Higher eHealth literacy significantly improved facilitating conditions (*β*=0.323; *P*<.001), self-efficacy (*β*=0.204; *P*<.001), performance expectancy (*β*=0.097; *P*=.04), and effort expectancy (*β*=0.164; *P*<.001), supporting H18-H21. However, it did not significantly reduce perceived risk (*β*=−0.063; *P*=.21), leading to rejection of H23. Notably, eHealth literacy increased distrust (*β*=0.150; *P*=.02), yielding a statistically significant but directionally reversed effect relative to H22. These findings suggest that individuals with higher digital health competencies may develop a more critical awareness of potential risks and limitations in digital health systems.

Perceived risk consistently undermined acceptance-related perceptions. It significantly increased distrust (*β*=0.265; *P*<.001), supporting H17, and negatively affected facilitating conditions (*β*=−0.112; *P*=.003), effort expectancy (*β*=−0.117; *P*=.002), and performance expectancy (*β*=−0.079; *P*=.04), supporting H14-H16. These findings indicate that privacy and security concerns simultaneously increase distrust while reducing perceived usefulness, ease of use, and infrastructural support for mHealth technologies.

Self-efficacy did not exhibit significant direct effects on the core UTAUT constructs in the overall model. Its paths to performance expectancy (*β*=0.100; *P*=.06), effort expectancy (*β*=−0.022; *P*=.67), and facilitating conditions (*β*=0.046; *P*=.37) were not significant, and therefore H11-H13 were not supported. These results suggest that self-efficacy primarily functions as an outcome of social influence and eHealth literacy rather than as a direct determinant.

**Table 4:** Structural path coefficients for overall sample (N=960). P values <.05 were considered statistically significant.

Category	Path	*β*	*P* value	Hypothesis	Result
BI^a^	EE^b^ → BI	0.404	<.001	H2	Supported
BI	PE^c^ → BI	0.188	<.001	H1	Supported
BI	FC^d^ → BI	0.349	<.001	H3	Supported
BI	DT^e^ → BI	–0.027	.45	H4	Not supported
SI^f^	SI → EE	0.811	<.001	H6	Supported
SI	SI → FC	0.548	<.001	H7	Supported
SI	SI → PE	0.662	<.001	H5	Supported
SI	SI → SE^g^	0.422	<.001	H8	Supported
SI	SI → eHL^h^	0.507	<.001	H9	Supported
SI	SI → DT	–0.248	<.001	H10	Supported
eHL	eHL → FC	0.323	<.001	H20	Supported
eHL	eHL → SE	0.204	<.001	H21	Supported
eHL	eHL → DT	0.15	.02	H22	Supported (opposite direction)
eHL	eHL → PE	0.097	.04	H18	Supported
eHL	eHL → PR^i^	–0.063	.21	H23	Not supported
eHL	eHL → EE	0.164	<.001	H19	Supported
PR	PR → DT	0.265	<.001	H17	Supported
PR	PR → FC	–0.112	.003	H16	Supported
PR	PR → EE	–0.117	.002	H15	Supported
PR	PR → PE	–0.079	.04	H14	Supported
SE	SE → PE	0.1	.06	H11	Not supported
SE	SE → EE	–0.022	.67	H12	Not supported
SE	SE → FC	0.046	.37	H13	Not supported
DT	DT → EE	0.003	.94	H25	Not supported
DT	DT → PE	–0.017	.70	H24	Not supported
DT	DT → FC	0.125	.006	H26	Not supported
Health-related covariates	Health status → PE	–0.016	.64	—^j^	—
Health-related covariates	Health interest → PE	0.133	<.001	—	—
Health-related covariates	Health status → DT	0.049	.33	—	—
Health-related covariates	Health interest → DT	–0.054	.29	—	—

^a^BI: behavioral intention.

^b^EE: effort expectancy.

^c^PE: performance expectancy.

^d^FC: facilitating conditions.

^e^DT: distrust.

^f^SI: social influence.

^g^SE: self-efficacy.

^h^eHL: eHealth literacy.

^i^PR: perceived risk.

^j^Not applicable.

Distrust showed limited direct effects. It did not significantly influence effort expectancy (*β*=0.003; *P*=.94) or performance expectancy (*β*=−0.017; *P*=.70); therefore, H25 and H24 were not supported. Contrary to H26, distrust showed a small but significant positive effect on facilitating conditions (*β*=0.125; *P*=.006), indicating that the predicted negative relationship was not confirmed; therefore, H26 was not supported. This unexpected direction suggests that distrust may prompt compensatory information-seeking behavior rather than withdrawal from support structures.

Health-related covariates showed selective effects. Health interest significantly increased performance expectancy (*β*=0.133; *P*<.001), indicating that individuals with greater health interest perceive higher utility in mHealth technologies. In contrast, self-rated health status did not significantly influence performance expectancy (*β*=−0.016; *P*=.64), and neither health interest nor health status significantly predicted distrust (*β*=−0.054; *P*=.29 and *β*=0.049; *P*=.33, respectively). These covariate effects were included for control purposes and were not part of the formal hypothesis testing framework.

### Sex-Stratified Analysis

The multigroup structural equation model for sex demonstrated acceptable to good model fit. The chi-square statistic was significant (*χ*^2^_904_=1994.9; *P*<.001), which is expected given the large sample size; however, the ratio of chi-square to degrees of freedom was within an acceptable range (*χ*^2^/df=2.21).

Incremental fit indices indicated good model fit (CFI=0.944; TLI=0.935; incremental fit index (IFI)=0.944). The RMSEA value was 0.035 (90% CI 0.033-0.038; *P*_close_>.99), indicating excellent fit. Although the GFI (0.882) and AGFI (0.853) were slightly below the conventional threshold, these indices are known to be sensitive to sample size and model complexity.

Overall, these results suggest that the model provides a satisfactory representation of the data and is appropriate for subsequent multigroup comparisons by sex.

The explanatory power of the model remained high across sex groups. The model explained a substantial proportion of variance in behavioral intention, accounting for 71.7% of the variance among males and 70.1% among females. Sex-stratified structural path coefficients are presented in Table 5.

First, social influence significantly reduced distrust among males (*β*=−0.325; *P*<.001) but not among females (*β*=−0.100; *P*=.16; Δ*χ*^2^=5.7; *P*=.02). This finding indicates that normative social cues function as more effective trust-building mechanisms for male users.

Second, eHealth literacy significantly reduced perceived risk among females (*β*=−0.210; *P*<.001) but not among males (*β*=−0.080; *P*=.11; Δ*χ*^2^=5.2; *P*=.02). This suggests that digital health competencies play a stronger role in mitigating privacy and security concerns among women.

Third, distrust exerted significantly stronger effects on effort expectancy among females (*β*=0.287; *P*<.001) than among males (*β*=0.031; *P*=.48; Δ*χ*^2^=17.3; *P*<.001). This indicates that distrust more strongly shapes perceived ease of use among female users.

Fourth, distrust also had a significantly stronger effect on performance expectancy among females (*β*=0.226; *P*<.001) than among males (*β*=−0.020; *P*=.65; Δ*χ*^2^=12.6; *P*<.001), suggesting that distrust influences perceived usefulness primarily among women.

Fifth, performance expectancy exerted significantly stronger effects on behavioral intention among females (*β*=0.388; *P*<.001) than among males (*β*=0.200; *P*<.001; Δ*χ*^2^=4.0; *P*=.04). These results indicate that perceived health benefits constitute a more central driver of mHealth adoption intentions for female users.

In contrast, no significant sex difference was observed for the path from distrust to behavioral intention (Δ*χ*^2^=1.7; *P*=.19), although the effect was statistically significant only among females.

**Table 5:** Sex-stratified path coefficients. Chi-square difference tests identified 5 statistically significant sex differences (Δχ2>3.8; P<.05).

Pathway and path direction				Male									Female									Chi-square difference (*df*); *P* value
				*β*		SE		T value		*P* value		*β*			SE		T value		*P* value			
SI^a^ pathways																						
	eHL^b^	SI → eHL	0.493		0.061		9.436		<.001		0.433			0.057		7.765		<.001		0.9 (1); .34		
	SE^c^	SI → SE	0.406		0.065		6.497		<.001		0.324			0.068		5.145		<.001		—^d^		
	DT^e^	SI → DT	–0.325		0.071		–4.287		<.001		–0.1			0.055		–1.395		.16		5.7 (1); .02		
	PE^f^	SI → PE	0.616		0.061		10.31		<.001		0.59			0.058		9.719		<.001		—		
	EE^g^	SI → EE	0.77		0.06		12.08		<.001		0.699			0.055		11.702		<.001		0.9 (1); .34		
	FC^h^	SI → FC	0.559		0.066		9.601		<.001		0.672			0.063		12.536		<.001		3.4 (1); .07		
eHL pathways																						
	SE	eHL → SE	0.22		0.052		3.802		<.001		0.264			0.062		4.452		<.001		—		
	PR	eHL → PR^i^	–0.08		0.061		–1.585		.11		–0.21			0.071		–4.029		<.001		5.2 (1); .02		
	DT	eHL → DT	0.135		0.052		2.081		.04		0.26			0.052		3.718		<.001		1.6 (1); .21		
	PE	eHL → PE	0.131		0.04		2.825		.005		0.097			0.049		1.823		.07		1.0 (1); .32		
	EE	eHL → EE	0.201		0.037		4.425		<.001		0.181			0.045		3.648		<.001		—		
	FC	eHL → FC	0.329		0.046		6.958		<.001		0.273			0.053		5.885		<.001		—		
PR pathways																						
	DT	PR → DT	0.259		0.037		4.676		<.001		0.178			0.032		3.004		.003		—		
	FC	PR → FC	–0.140		0.032		–3.540		<.001		–0.143			0.032		–3.821		<.001		—		
	EE	PR → EE	–0.118		0.026		–3.093		.002		–0.212			0.028		–5.08		<.001		1.7 (1); .20		
	PE	PR → PE	–0.054		0.028		–1.377		.17		–0.165			0.031		–3.647		<.001		0.7 (1); .40		
SE pathways																						
	DT	SE → DT	0.096		0.065		1.32		.19		0.013			0.051		0.181		.86		—		
	PE	SE → PE	0.142		0.05		2.737		.006		–0.027			0.048		–0.496		.62		—		
	EE	SE → EE	–0.007		0.045		–0.139		.89		0.017			0.043		0.342		.73		—		
	FC	SE → FC	0.044		0.055		0.954		.34		–0.029			0.051		–0.628		.53		—		
DT pathways																						
	EE	DT → EE	0.031		0.045		0.705		.48		0.287			0.06		5.795		<.001		17.3 (1); <.001		
	FC	DT → FC	0.129		0.055		2.841		.004		0.102			0.064		2.481		.01		—		
	PE	DT → PE	–0.020		0.049		–0.453		.65		0.226			0.064		4.414		<.001		12.6 (1); <.001		
BI^j^ pathways																						
	BI	PE → BI	0.2		0.064		3.881		<.001		0.388			0.067		7.362		<.001		4.0 (1); .04		
	BI	FC → BI	0.336		0.062		5.998		<.001		0.177			0.068		2.652		.008		1.4 (1); .23		
	BI	EE → BI	0.358		0.087		5.43		<.001		0.325			0.103		4.109		<.001		0.6 (1); .45		
	BI	DT → BI	–0.051		0.048		–1.404		.16		–0.102			0.065		–2.486		.01		1.7 (1); .19		
Health-related covariates																						
	PE	health status → PE	–0.017		0.022		–0.495		.62		–0.063			0.022		–1.702		.09		—		
	DT	health status → DT	0.049		0.03		0.957		.34		–0.136			0.026		–2.608		.009		—		
	PE	health interest → PE	0.132		0.023		3.785		<.001		0.172			0.027		4.608		<.001		—		
	DT	health interest → DT	–0.053		0.031		–1.044		.30		–0.014			0.03		–0.27		.79		—		

^a^SI: social influence.

^b^eHL: eHealth literacy.

^c^SE: self-efficacy.

^d^Not applicable.

^e^DT: distrust.

^f^PE: performance expectancy.

^g^EE: effort expectancy.

^h^FC: facilitating conditions.

^i^PR: perceived risk.

^j^BI: behavioral intention.

### Age-Stratified Analysis

For age-stratified multigroup SEM, participants were categorized into 3 groups: young adults (aged 18-39 years; n=298), middle-aged adults (aged 40-59 years; n=275), and older adults (aged ≥60 years; n=387).

The multigroup structural equation model across the 3 age groups demonstrated acceptable to good fit: *χ*^2^_1356_=2762.4; *P*<.001; *χ*^2^/*df*=2.04; GFI=0.849; AGFI=0.812; normed fit index 0.871; CFI=0.929; TLI=0.917; IFI=0.930; RMSEA=0.033 (90% CI 0.031-0.035; *P*_close_>.99).

Although GFI and AGFI were slightly below the conventional threshold of 0.90, these indices are known to be sensitive to model complexity and sample size. In contrast, incremental fit indices (CFI, TLI, and IFI) exceeded recommended thresholds, and RMSEA indicated excellent model fit. Taken together, these results suggest that the age-stratified model provides an adequate and robust representation of the observed data.

The explanatory power of the model remained high across age groups. The model explained 77.1% of the variance in behavioral intention among young adults, 67.5% among middle-aged adults, and 69.1% among older adults (Table 6).

First, social influence exerted significantly different effects on performance expectancy across age groups (Δ*χ*^2^_2_=11.76; *P*<.01). The effect was strongest among young adults (*β*=0.919; *P*<.001), followed by middle-aged adults (*β*=0.617; *P*<.001) and older adults (*β*=0.574; *P*<.001), indicating that social cues play a more prominent role in shaping perceived usefulness among younger individuals.

Second, eHealth literacy showed significant age differences in its effect on perceived risk (Δ*χ*^2^_2_=13.7; *P*<.01). Specifically, eHealth literacy significantly reduced perceived risk among middle-aged adults (*β*=−0.187; *P*=.005) and older adults (*β*=−0.231; *P*<.001), whereas no significant association was observed among young adults (*β*=0.061; *P*=.36). This suggests that digital health competencies function as a risk-mitigating factor primarily in older populations.

Third, self-efficacy exhibited the largest age-related heterogeneity in its effect on performance expectancy (*Δχ*^2^_2_=17.1; *P*<.001). Notably, self-efficacy negatively influenced performance expectancy among young adults (*β*=−0.324; *P*<.001), while showing positive effects among middle-aged adults (*β*=0.161; *P*=.02) and older adults (*β*=0.110; *P*=.05). This reversal suggests that confidence in one’s digital abilities may lead to more critical evaluations of mHealth usefulness among younger users.

Fourth, distrust demonstrated significant age differences in its effect on effort expectancy (Δ*χ*^2^_2_=13.5; *P*<.01). Distrust strongly increased perceived difficulty of use among young adults (*β*=0.349; *P*<.001), whereas this relationship was not significant among middle-aged adults (*β*=0.075; *P*=.22) or older adults (*β*=0.039; *P*=.37).

Fifth, distrust also showed significant age differences in its effect on performance expectancy (Δ*χ*^2^_2_=10.7; *P*<.01). Distrust positively influenced performance expectancy among young adults (*β*=0.249; *P*<.001), while no significant effects were observed among middle-aged adults (*β*=−0.002; *P*=.97) or older adults (*β*=0.022; *P*=.66), indicating divergent interpretations of distrust across age groups.

In contrast, no significant age differences were observed in the effects of performance expectancy (Δ*χ*^2^_2_=1.7; *P*>.05), facilitating conditions (Δ*χ*^2^_2_=3.6; *P*>.05), or distrust (Δ*χ*^2^_2_=9.1; *P*>.01) on behavioral intention, and age differences in the direct effect of distrust on behavioral intention were confined to middle-aged adults. Thus, while distrust showed some age-contingent influence on intention, the core positive determinants of mHealth adoption (performance expectancy, effort expectancy, and facilitating conditions) remained largely invariant across age groups.

These findings indicate that the proximal determinants of mHealth adoption intention are largely invariant across age groups, despite substantial heterogeneity in upstream cognitive and perceptual mechanisms. Health-related covariates showed a consistent pattern across age groups. Health interest significantly increased performance expectancy in all 3 age groups (young adults: *β*=0.133; *P*=.002; middle-aged adults: *β*=0.090; *P*=.05; older adults: *β*=0.202; *P*<.001), with the strongest effect among older adults, whereas self-rated health status did not significantly predict performance expectancy or distrust in any group.

**Table 6:** Age-stratified path coefficients. Chi-square difference tests identified 5 statistically significant age differences (Δχ2>6.0; P<.05).

Pathway and path direction		Young adults				Middle-aged adults				Older adults				Age, chi-square difference (*df*); *P* value
		*β*	T value	*P* value	*β*		T value	*P* value	*β*		T value	*P* value		
SI^a^ pathways														
	eHL^b^ ← SI	0.593	7.698	<.001	0.31		4.459	<.001	0.456		7.437	<.001	—^c^	
	DT^d^ ← SI	–0.234	–2.285	.02	–0.215		–2.676	.007	–0.159		–2.228	.03	—	
	SE^e^ ← SI	0.602	6.286	<.001	0.353		4.798	<.001	0.229		3.273	.001	—	
	PE^f^ ← SI	0.919	8.165	<.001	0.617		8.86	<.001	0.574		9.026	<.001	11.8 (2); <.01	
	EE^g^ ← SI	1.03	8.219	<.001	0.745		10.11	<.001	0.707		11.738	<.001	—	
	FC^h^ ← SI	0.599	6.62	<.001	0.626		9.581	<.001	0.651		11.115	<.001	—	
eHL pathways														
	PR^i^ ← eHL	0.061	0.912	.36	–0.187		–2.79	.005	–0.231		–4.14	<.001	13.7 (2); <.01	
	DT ← eHL	0.366	3.664	<.001	0.119		1.513	.13	0.089		1.274	.20	—	
	SE ← eHL	0.166	2.041	.04	0.288		4.055	<.001	0.243		3.601	<.001	—	
	PE ← eHL	0.103	1.31	.19	0.09		1.551	.12	0.101		1.815	.07	—	
	EE ← eHL	–0.009	–0.11	.91	0.217		3.62	<.001	0.25		5.015	<.001	—	
	FC ← eHL	0.322	4.51	<.001	0.339		6.011	<.001	0.209		4.045	<.001	—	
PR pathways														
	DT ← PR	0.153	2.116	.03	0.25		3.303	<.001	0.186		3.026	.002	—	
	FC ← PR	–0.108	–2.202	.03	–0.234		–4.77	<.001	–0.126		–2.951	.003	—	
	EE ← PR	–0.16	–2.948	.003	–0.098		–1.832	.07	–0.139		–3.444	<.001	—	
	PE ← PR	–0.184	–3.53	<.001	–0.114		–2.134	.03	0.009		0.186	.85	—	
Self–efficacy pathways														
	PE ← SE	–0.324	–3.384	<.001	0.161		2.399	.02	0.11		1.998	.05	17.1 (2); <.001	
	EE ← SE	–0.222	–2.271	.02	0.089		1.331	.18	0.061		1.307	.19	—	
	FC ← SE	–0.024	–0.305	.76	0.027		0.45	.65	0.014		0.275	.78	—	
Distrust pathways														
	EE ← DT	0.349	4.906	<.001	0.075		1.225	.22	0.039		0.899	.37	13.5 (2); <.01	
	FC ← DT	0.204	3.541	<.001	0.036		0.67	.50	0.09		1.985	.05	—	
	PE ← DT	0.249	3.94	<.001	–0.002		–0.036	.97	0.022		0.438	.66	10.7 (2); <.01	
BI^j^ pathways														
	BI ← EE	0.377	3.259	.001	0.231		2.301	.02	0.419		5.63	<.001	1.5 (2); .48	
	BI ← PE	0.272	3.16	.002	0.216		3.025	.002	0.284		5.299	<.001	1.7 (2); .43	
	BI ← FC	0.352	4.158	<.001	0.42		4.595	<.001	0.254		3.964	<.001	3.6 (2); .17	
	BI ← DT	–0.113	–2.209	.03	–0.171		–3.159	.002	0.028		0.702	.48	9.1 (2); .01	
Health-related covariates														
	PE ← health status	–0.039	–0.911	.36	–0.043		–0.925	.36	–0.022		–0.53	.60	—	
	DT ← health status	–0.09	–1.39	.16	–0.009		–0.126	.90	–0.04		–0.713	.49	—	
	PE ← health interest	0.133	3.126	.002	0.09		1.957	.05	0.202		4.707	<.001	—	
	DT ← health interest	0.017	0.268	.79	–0.029		–0.423	.67	0.091		1.634	.10	—	

^a^SI: social influence.

^b^eHL: eHealth literacy.

^c^Not applicable.

^d^DT: distrust.

^e^SE: self-efficacy.

^f^PE: performance expectancy.

^g^EE: effort expectancy.

^h^FC: facilitating conditions.

^i^PR: perceived risk.

^j^BI: behavioral intention.

### App-Specific Use Patterns: Binary Logistic Regression Analysis

Binary logistic regression analysis examined predictors of current use across 3 app types: blood pressure monitoring (n=173), heart rate monitoring (n=118), and physical activity tracking (n=110), using the original 7 age strata as categorical predictors. All models demonstrated adequate fit (Hosmer-Lemeshow *P*>.05) with a Nagelkerke *R*^2^ of 0.14 for each model. Table 7 presents ORs and 95% CIs.

**Table 7:** App-specific predictors of current mHealth use.

Predictor		Blood pressure (n=173)	Heart rate (n=118)	Physical activity (n=110)
Behavioral constructs, OR^a^ (95% CI)				
	Behavioral intention	1.40 (1.08-1.80)^b^	1.77 (1.30-2.41)^c^	2.47 (1.76-3.47)^c^
	eHealth literacy	1.85 (1.42-2.42)^c^	1.84 (1.35-2.53)^c^	1.57 (1.14-2.17)^d^
Demographics, OR (95% CI)				
	Sex (Male; ref)	0.74 (0.52-1.05)	0.89 (0.59-1.33)	0.82 (0.54-1.25)
Age group (years; ref=18-27), OR (95% CI)				
	28-37	0.64 (0.29-1.40)	0.62 (0.27-1.44)	0.97 (0.44-2.13)
	38-47	0.88 (0.42-1.86)	1.21 (0.57-2.59)	1.23 (0.57-2.65)
	48-57	1.25 (0.61-2.56)	0.94 (0.41-2.11)	1.58 (0.74-3.40)
	58-67	2.34 (1.18-4.61)^b^	1.26 (0.57-2.77)	1.03 (0.45-2.39)
	68-77	3.15 (1.63-6.12)^c^	2.37 (1.15-4.89)^b^	1.59 (0.74-3.45)
	≥78 years	3.76 (1.93-7.32)^c^	1.91 (0.90-4.08)	1.06 (0.46-2.45)
Model fit				
	Hosmer-Lemeshow *χ*^2^(*df*); *P* value	6.92 (8); .55	3.55 (8); .90	7.76 (8); .46

^a^OR: odds ratio.

^b^*P*<.05.

^c^*P*<.001.

^d^*P*<.01.

Behavioral intention and eHealth literacy emerged as consistent significant predictors across all 3 app types. For blood pressure monitoring, behavioral intention (OR 1.40, 95% CI 1.08-1.80; *P*=.01) and eHealth literacy (OR 1.85, 95% CI 1.42-2.42; *P*<.001) significantly predicted current use. Sex was not a significant predictor (OR 0.74, 95% CI 0.52-1.05). Age demonstrated a pronounced gradient compared with users aged 18-27 years, those aged 58-67 years (OR 2.34, 95% CI 1.18-4.61; *P*=.02), aged 68-77 years (OR 3.15, 95% CI 1.63-6.12; *P*<.001), and aged ≥78 years (OR 3.76, 95% CI 1.93-7.32; *P*<.001) exhibited substantially elevated odds of use, reflecting the clinical relevance of hypertension management among older populations.

For heart rate monitoring, behavioral intention (OR 1.77, 95% CI 1.30-2.41; *P*<.001) and eHealth literacy (OR 1.84, 95% CI 1.35-2.53; *P*<.001) significantly predicted current use. Sex was not significant (OR 0.89, 95% CI 0.59-1.33). Age effects were less pronounced than for blood pressure monitoring, with only users aged 68-77 years demonstrating significantly elevated odds (OR 2.37, 95% CI 1.15-4.89; *P*=.02), suggesting broader age-range appeal for heart rate monitoring compared with blood pressure monitoring.

Physical activity tracking demonstrated the strongest behavioral intention effect among the 3 apps (OR 2.47, 95% CI 1.76-3.47; *P*<.001), with eHealth literacy also significantly predicting use (OR 1.57, 95% CI 1.14-2.17; *P*=.006). Sex (OR 0.82, 95% CI 0.54-1.25) and all age groups showed no significant differences compared with the reference group, indicating cross-demographic adoption patterns driven by intrinsic motivation and digital health competency rather than age-related clinical necessity.

## Discussion

### Overview

This study examined the determinants of mHealth acceptance in Japan using an extended UTAUT framework that incorporated distrust, perceived risk, and eHealth literacy, while additionally modeling health status and health interest as covariates of performance expectancy and distrust. Overall, the findings confirmed the central role of the core UTAUT constructs—performance expectancy, effort expectancy, and facilitating conditions—in shaping behavioral intention, while also demonstrating that demographic heterogeneity in Japan is concentrated primarily in upstream cognitive and evaluative processes rather than in the final intention-formation stage [20-22,50,52,54,55]. This distinction is important because it suggests that the basic decision logic of mHealth acceptance is relatively stable, even in a superaged society, whereas the pathways through which individuals form beliefs about usefulness, ease of use, trust, and risk differ substantially by age and sex.

### Principal Findings and Theoretical Contributions

Consistent with prior UTAUT-based research, performance expectancy, effort expectancy, and facilitating conditions emerged as robust predictors of behavioral intention in the overall model [20-22,50,52,54,55,79]. This pattern is also broadly consistent with earlier studies on mHealth adoption among older adults and chronic disease populations [23,24,28,29]. However, this study extends this literature by showing that, although these proximal determinants remain central, age differences were not statistically significant for the direct effects of performance expectancy and facilitating conditions on behavioral intention. This suggests that the core decision structure linking beliefs to intention may be more stable across the life course than is often assumed. Put differently, age appears to matter less for how people translate beliefs into intention than for how those beliefs are initially formed.

A major theoretical contribution of this study is the expanded role of social influence. In conventional UTAUT, social influence is usually modeled as one determinant of intention or belief formation [20,21]. In this study, however, social influence exerted broad and upstream effects on eHealth literacy, self-efficacy, performance expectancy, effort expectancy, and facilitating conditions, while also reducing distrust. This broader role exceeds many Western UTAUT applications [20,22] and is consistent with the Japanese context, where family members, peers, and health care professionals often function as key agents of reassurance, persuasion, and health-related decision support [18,19,27]. The age-stratified analyses further showed that the effect of social influence on performance expectancy was strongest among young adults. This finding suggests that, in contemporary Japan, younger users may evaluate the usefulness of mHealth partly through socially mediated expectations and peer-based information environments rather than solely through individual instrumental reasoning. In this sense, social influence in Japan may operate not only as a normative force but also as a mechanism of informal digital health education.

A second major contribution concerns eHealth literacy. Previous studies have generally positioned eHealth literacy as a uniformly beneficial facilitator of digital health use [16,17,45,46,64,80-82]. In Japan, the Japanese eHealth Literacy Scale has demonstrated strong internal consistency and construct validity in a large internet-based survey, with higher scores among middle-aged adults and frequent internet users [83]. Our results support this view only partially. Among middle-aged and older adults, higher eHealth literacy significantly reduced perceived risk, which is consistent with both international research and Japanese evidence linking eHealth literacy to improved health knowledge and screening-related practices [16,17,64]. However, this pattern did not extend to younger adults. In the younger group, eHealth literacy did not reduce perceived risk and was accompanied by more critical appraisals in related pathways. This suggests that eHealth literacy is better conceptualized as a context-dependent cognitive resource: it may reduce uncertainty in populations with lower baseline digital familiarity, but among digitally experienced users, it may instead sharpen awareness of privacy vulnerability, data misuse, or functional limitations. This interpretation aligns with research on the privacy paradox and digitally engaged patients [12,25,47,84,85].

A third theoretical contribution lies in the age-differentiated role of self-efficacy. In many prior studies, self-efficacy has shown positive associations with perceived usefulness and ease of use [20,57,60,61]. In this study, however, self-efficacy did not significantly predict performance expectancy, effort expectancy, or facilitating conditions in the overall model, and age-stratified analyses revealed a more complex pattern. Most notably, self-efficacy negatively predicted performance expectancy among younger adults but positively predicted it among middle-aged and older adults. This reversal suggests that digitally confident younger users may hold higher expectations for digital services and therefore evaluate mHealth apps more critically when the apps fail to meet their standards, whereas among middle-aged and older adults, self-efficacy plays the more conventional enabling role by lowering psychological barriers and making benefits more salient [23,26,28]. At the same time, the weak direct effect of self-efficacy in the overall model and its conceptual proximity to eHealth literacy indicate that self-efficacy in mHealth acceptance may operate more indirectly and contextually than previous models assume, partly overlapping with eHealth literacy as a mediating cognitive resource [16,17,64,82].

A fourth contribution is the clearer differentiation between distrust and perceived risk. Prior information systems and digital health research have often linked trust, distrust, and risk closely, but not always modeled them as distinct pathways [12-15,30,39,42,58,59,62,63]. In this study, perceived risk and distrust behaved differently. Perceived risk consistently undermined acceptance-related beliefs, whereas distrust showed selective and demographically contingent effects. In the overall model, distrust did not significantly reduce behavioral intention, but it influenced effort expectancy and performance expectancy in subgroup analyses, particularly among women and younger adults. These findings suggest that distrust should not be reduced to a mere proxy for perceived risk. Rather, distrust appears to function as a broader evaluative orientation toward the technology, platform, or data environment, while perceived risk reflects a more specific anticipation of privacy or security harm [14,30,58,59]. This conceptual separation is valuable for digital health research, where users may simultaneously recognize objective risks and still maintain—or fail to maintain—confidence in the system’s legitimacy and safety [12,13,40-43,86,87]. Consistent with the additional reduced-item models, the selective and mainly indirect role of distrust appears to be driven more by its structural position in the extended UTAUT framework than by weaknesses in the measurement model, reinforcing the need to interpret distrust as a nuanced, context-dependent barrier rather than a uniformly dominant deterrent to mHealth adoption.

Contrary to H26, distrust showed a small but significant positive effect on facilitating conditions. This unexpected finding may reflect compensatory information-seeking behavior, whereby skeptical users actively engage with available support structures to verify system safety rather than withdrawing from them [88]. In addition, the strong role of social influence and the differentiated effects of distrust are congruent with culturally specific notions such as sekentei (concern for social evaluation) and anshin (sense of safety and peace of mind), which shape how Japanese users balance institutional assurances, peer endorsement, and perceived vulnerability when deciding whether to adopt mHealth services [89,90].

### Sex and Age Heterogeneity in the Japanese Context

The sex-stratified results revealed systematic, rather than incidental, heterogeneity. Compared with women, men showed a stronger negative pathway from social influence to distrust, suggesting that social endorsement and interpersonal reassurance play a larger role in trust formation among male users. By contrast, women showed a stronger negative pathway from eHealth literacy to perceived risk and a stronger positive pathway from performance expectancy to behavioral intention. These findings are broadly consistent with prior research suggesting that women are more attentive to privacy, risk, and benefit evaluation in digital contexts, whereas men may rely more heavily on social and structural cues [31,79,90]. In the Japanese context, this implies that male users may respond particularly well to social endorsement and visible legitimacy, whereas female users may be more influenced by the extent to which a system appears beneficial, transparent, and safe.

The age-stratified results were especially informative. Significant age differences were identified in the pathways from social influence to performance expectancy, eHealth literacy to perceived risk, self-efficacy to performance expectancy, and distrust to both effort expectancy and performance expectancy. In contrast, no statistically significant age differences were observed in the direct effects of performance expectancy or facilitating conditions on behavioral intention. This pattern strongly suggests that age-related heterogeneity in Japan is concentrated in the formation of beliefs rather than in the final translation of those beliefs into adoption intention.

Among young adults, social influence had the strongest effect on performance expectancy, and distrust significantly affected both effort expectancy and performance expectancy. This suggests that younger users do not simply accept or reject mHealth technologies based on distrust alone; rather, distrust may stimulate more active evaluation of usability and utility. Such a pattern is compatible with the idea of “informed skepticism,” in which digitally experienced users are not necessarily more trusting, but instead more critical and selective [12,47,79,84]. Among middle-aged adults, eHealth literacy reduced perceived risk, and self-efficacy positively influenced performance expectancy, suggesting a more instrumental and balanced evaluative style. This may reflect the life-stage demands of work, family responsibility, and health management, which make reliability and usefulness especially salient. This interpretation is also consistent with recent Japanese evidence showing that mHealth adoption among workers is associated with health-related motivations and daily-life relevance [7]. Among older adults, eHealth literacy also reduced perceived risk, but distrust no longer affected behavioral intention. This suggests that older adults may rely more on literacy-based reassurance and practical value than on distrust-sensitive rejection. Rather than indicating a simple deficit, this pattern implies a functional reorganization of evaluation in later life, consistent with earlier studies emphasizing usability support, family assistance, and the need for age-sensitive design [9,23,26,28,82,91].

Taken together, these findings help explain a persistent Japanese paradox. Japan is characterized by high life expectancy, universal health coverage, extensive digital infrastructure, and rapid population aging, yet mHealth adoption remains limited [3-7]. Our results suggest that this underuse is not explained simply by a lack of infrastructure or a universal failure of perceived usefulness. Rather, it stems from demographic differences in how usefulness, trust, literacy, and risk are interpreted and weighted. In that sense, this study extends prior Japanese work by moving from a descriptive discussion of barriers to a structural account of how those barriers are organized within the acceptance process.

### Health-Related Covariates and Contextual Interpretation

An important addition prompted by the revision process was the inclusion of health status and health interest as covariates of performance expectancy and distrust. These analyses strengthened the interpretation of the model in meaningful ways. Health interest positively predicted performance expectancy in the overall model and across sex and age subgroups, with particularly strong effects among older adults. This indicates that users who are more concerned about their health are more likely to perceive mHealth technologies as beneficial. This result is intuitively plausible and aligns with earlier research suggesting that health motivation is a key antecedent of engagement with preventive and self-management applications [1,36,37].

By contrast, self-rated health status showed weaker and less consistent effects. It did not significantly predict performance expectancy in the overall model or across most subgroups, although in some subgroup analyses it was associated with lower distrust. These findings suggest that subjective health concern may be a more important driver of acceptance-related beliefs than objective or self-perceived health condition per se. This is a useful refinement of the “clinical necessity” argument: it is not simply being less healthy that matters, but being more attentive to one’s health and more motivated to manage it. Accordingly, claims about “pragmatic acceptance” among older adults should be interpreted in relation to health interest and literacy rather than assumed solely from age or illness burden.

### App-Type Specificity

The app-type-specific analyses further emphasized that mHealth adoption is not monolithic. Disease-management apps were more strongly associated with older adults and with contexts of clinical necessity, consistent with prior telemonitoring and chronic disease management studies [2,37,80,81,92,93]. By contrast, wellness and physical activity apps showed broader cross-demographic appeal and appeared to be driven more by behavioral engagement than by medical need [1,36,37]. This distinction is important because it implies that “performance expectancy” may not mean the same thing across app categories. In disease management, perceived usefulness is likely tied to symptom control, physician recommendation, and safety; in wellness apps, it may reflect motivation, convenience, enjoyment, or self-optimization [1,36,44,56]. Future models of mHealth acceptance should therefore be more explicit about app context rather than assuming that adoption mechanisms are uniform across all digital health tools.

### Practical Implications

Several practical implications follow. First, trust-centered strategies are essential. Because distrust and perceived risk operate differently across demographic groups, improving app functionality alone will not be sufficient. Transparent data governance, visible compliance with privacy regulations, third-party security certification, and clear communication about data use are especially important in the Japanese setting, where public sensitivity to personal information protection remains high [12,13,25,40,41,86,87].

Second, interventions should combine universal and targeted strategies. Universal approaches can focus on improving performance expectancy, effort expectancy, and facilitating conditions, because these remain stable predictors of intention across age groups. However, upstream mechanisms require tailoring. For younger users, transparent governance and user control may be more effective than generic promotional messages. For middle-aged users, interventions should emphasize practical value, reliability, and low-risk integration into everyday life. Even among digitally reachable older adults with relatively high eHealth literacy, digital literacy support, simplified interfaces, and family- or community-assisted onboarding remain important for sustaining engagement, while interventions targeting offline or low-connectivity seniors will require additional outreach and support mechanisms [9,23,26,28,82].

Third, implementation in Japan should make explicit use of interpersonal and institutional actors. The structural breadth of social influence observed in this study indicates that peers, family members, workplaces, and health care professionals are not merely dissemination channels but active components of the acceptance process [18,19,27]. This has implications for public health campaigns, workplace wellness programs, and physician-endorsed disease management systems.

### Limitations and Future Research

Several limitations should be noted. First, the study relied on an online panel, which likely underrepresents individuals with limited internet access or lower digital engagement, especially among older adults [68,94]. Accordingly, the older group in this study should be interpreted primarily as digitally reachable older adults rather than as fully representative of the Japanese older population as a whole. In our sample, the mean eHealth literacy score was 22.84 (SD 6.31), which is somewhat higher than the 20.46 (SD 5.64) reported in a recent Japanese internet-based study using the eHealth Literacy Scale in a public health context [83]. Because both studies relied on online samples, these values likely overestimate eHealth literacy in the broader Japanese population, particularly among offline or low-connectivity seniors. Relative to the broader Japanese population, nondigital seniors may have lower eHealth literacy, greater usability barriers, and stronger dependence on facilitating conditions, meaning that the present findings may underestimate acceptance barriers in the most vulnerable groups. Future research should therefore use mixed-mode survey designs and community-based recruitment strategies—such as outreach through local clinics, senior centers, and home-visit services—to reach digitally excluded older adults who are absent from online panels and to better capture their acceptance mechanisms and support needs. Although our overall structural model showed acceptable fit according to incremental and parsimony-adjusted indices (CFI=0.953; TLI=0.945; RMSEA=0.047), the GFI (0.889) and AGFI (0.863) fell slightly below the conventional 0.90 threshold specified in the Methods section. Given the well-performing CFI, TLI, and RMSEA values and the known sensitivity of GFI and AGFI to sample size and model complexity, we interpret the structural model as adequately fitting while acknowledging this limitation.

Second, the cross-sectional design precludes causal inference and limits our ability to distinguish age-related differences from cohort or period effects [95]. Third, although the model explained a substantial proportion of variance in behavioral intention, future research should incorporate actual usage behavior and longitudinal follow-up to distinguish initial intention from sustained engagement [1,36,44,56]. Fourth, while this study identified meaningful age- and sex-related heterogeneity, future work should continue to test measurement robustness and explore whether similar structural patterns hold in other East Asian contexts or in more clinically specific populations [32-35,77,78]. Finally, because habit and hedonic motivation were not included, future research focusing on established users should examine whether UTAUT2 constructs become more relevant in later stages of mHealth engagement [50,56].

### Conclusion

This study provides robust empirical evidence that mHealth acceptance in Japan’s aging society is shaped by a dual structure consisting of stable proximal determinants and heterogeneous upstream belief-formation processes. While performance expectancy, effort expectancy, and facilitating conditions consistently predict behavioral intention across demographic groups, age and sex differences emerge primarily in how these beliefs are constructed through social influence, eHealth literacy, distrust, and perceived risk.

By extending the UTAUT framework to incorporate distrust, perceived risk, and eHealth literacy, and by integrating health status and health interest as contextual covariates, this study advances the theoretical understanding of digital health acceptance beyond conventional models. In particular, the findings demonstrate that eHealth literacy functions as a context-dependent cognitive resource, self-efficacy exhibits age-contingent effects—including a reversal pattern among younger users—and distrust operates through distinct mechanisms that are not reducible to perceived risk. These results challenge the assumption of uniform acceptance mechanisms and highlight the importance of distinguishing between belief formation and intention formation.

From a policy and practice perspective, the findings indicate that universal promotion strategies for mHealth are insufficient in aging societies. Instead, effective implementation requires trust-centered, demographically tailored, and context-sensitive approaches. For younger users, strategies should address informed skepticism through transparency and user control; for middle-aged users, emphasis should be placed on practical value and risk mitigation; and for older adults, interventions should prioritize literacy support, usability, and social or familial facilitation.

In the Japanese context—characterized by rapid population aging, advanced digital infrastructure, and strong institutional health care systems—these differentiated mechanisms help explain the persistent gap between technological availability and actual mHealth use. Addressing this gap requires not only improving technological functionality but also strengthening trust, enhancing digital health literacy, and leveraging social and institutional support structures.

Overall, this study contributes to the growing literature on digital health by demonstrating that mHealth acceptance is fundamentally shaped by the interaction of psychological, social, and demographic factors. Future research should further examine longitudinal adoption patterns, incorporate behavioral usage data, and explore culturally specific constructs to refine theory and inform more effective digital health interventions in aging societies.

## Data Availability

The datasets generated or analyzed during this study are available from the corresponding author on reasonable request.

## Appendix

Multimedia Appendix 1 CHERRIES (Checklist for Reporting Results of Internet E-Surveys) checklist.

Multimedia Appendix 2 Construct items and descriptive statistics (mean and SD).

Multimedia Appendix 3 Descriptive statistics and correlations.

Multimedia Appendix 4 Supplementary Figure 1. Usage rates of various mobile health applications.

Multimedia Appendix 5 Confirmatory factor analysis results: factor loadings, reliability, and validity indices for constructs and items.

## Abbreviations

AGFI: adjusted GFI
AVE: average variance extracted
CFA: confirmatory factor analysis
CFI: comparative fit index
CHERRIES: Checklist for Reporting Results of Internet E-Surveys
CR: composite reliability
GFI: goodness-of-fit index
IFI: incremental fit index
mHealth: mobile health
OR: odds ratio
RMSEA: root mean square error of approximation
SEM: structural equation modeling
TLI: Tucker-Lewis index
UTAUT: unified theory of acceptance and use of technology
