# Supplementary material for: Mobile Health App Acceptance in Japan’s Aging Society: Multigroup Structural Equation Modeling Based on the Extended Unified Theory of Acceptance and Use of Technology and eHealth Literacy Frameworks
Source: JMIR Mhealth Uhealth. 2026 Jun 9;14:e87832. doi: 10.2196/87832 (PMC13291735; doi:10.2196/87832)
Supplement: Multimedia Appendix 1 [file mhealth_v14i1e87832_app1.pdf]

Appendix CHERRIES Checklist

Item Category & Checklist Item	Description in This Study
Design - Target population	The survey targeted adults aged 18 years or older living in Japan. Participants were recruited from Cross Marketing Inc.'s pre-registered panel. To ensure demographic representativeness, stratified random sampling was applied by age (seven categories: 18–27, 28–37, 38–47, 48–57, 58–67, 68–77, and ≥78 years) and gender.
Design - Sampling frame	Cross Marketing Inc. invited individuals from its nationwide research panel who met inclusion criteria (≥18 years old, registered demographic information consistent with stratification categories). Screening questions (age, gender) were used to confirm eligibility, but exact inclusion criteria were not disclosed to participants.
Design – Sample	A total of 23,434 invitations were distributed. Among them, 2,203 participants provided informed consent, and after applying exclusion criteria (mismatch in registered vs. reported age/gender, extremely short response time, inconsistent or implausible answers, failure to follow instructions), 1,000 valid responses were retained for analysis.
IRB approval and informed consent process - IRB approval	Ethical approval was granted by the Waseda University Ethics Committee (Application No. 2023-250).
IRB approval and informed consent process - Informed consent	Before beginning the questionnaire, participants were provided with a detailed description of the study, including assurances that responses would be anonymized and that personally identifiable information would not be collected. Only those who clicked the

	consent confirmation button proceeded to the survey.
IRB approval and informed consent process - Data protection	No personally identifiable information was collected. All responses were anonymized, and non-eligible or non-consenting participants' data were immediately excluded.
Development and pretesting	The questionnaire was developed with reference to prior research on health data sharing, biobanks, and electronic medical records. Five academic staff and researchers conducted a pilot test between October 28 and November 6, 2023, to evaluate clarity, usability, and response burden. The final survey instrument was refined accordingly.
Recruitment - Open vs. closed survey	This was a closed survey. Only pre-registered monitors of Cross Marketing Inc. were invited.
Recruitment - Contact mode	Eligible panel members received invitations by email.
Recruitment – Advertising	No public advertisement was used; recruitment was restricted to the survey firm's panel.
Survey administration - Web/E-mail	The survey was conducted via a dedicated web-based platform managed by Cross Marketing Inc. Responses were automatically saved as participants navigated through pages.
Survey administration – Context	The survey site was hosted on a secure platform, independent of other websites, accessible only via the unique link provided in the invitation email.
Survey administration - Mandatory/voluntary	Participation was voluntary. Participants could withdraw at any stage prior to final submission.

Survey administration – Incentives	Panelists received reward points with monetary value according to the firm's standard incentive system.
Survey administration - Time/Date	The pilot survey was conducted between October 28 and November 6, 2023. The final survey was conducted from November 11 to 18, 2023.
Survey administration - Randomization of items	The order of questions was randomized to reduce response bias.
Survey administration - Adaptive questioning	Not applied. All participants received the same questions.
Survey administration - Number of items	The questionnaire contained domains covering five predictor categories (sociodemographic factors, types of health data shared, motivation for sharing, concerns regarding data sharing, reasonable access/control).
Survey administration - Number of screens/pages	The questionnaire was divided into multiple pages. Responses were saved automatically when participants moved to the next page.
Survey administration - Completeness check	All items were mandatory. A warning was displayed for missing responses, preventing participants from proceeding without completing each page.
Survey administration - Review step	Participants were able to revise their answers until final submission.
Response rates - Unique site visitors	Not applicable, as invitations were distributed only to pre-registered monitors with unique IDs.
Response rates - View rate	Not calculated.
Response rates - Participation rate	Of 23,434 invited participants, 2,203 provided consent (9.4%).

Response rates - Completion rate	Of 2,203 consenting participants, 1,000 provided valid responses after exclusions (4.3% of distributed).
Preventing multiple entries - Cookies used	Not used.
Preventing multiple entries - IP check	The system checked for duplicate entries, and suspicious responses (e.g., implausibly short completion times) were excluded.
Preventing multiple entries - Log file analysis	Not used.
Preventing multiple entries – Registration	All participants were pre-registered members of Cross Marketing Inc.'s survey panel.
Analysis - Handling of incomplete questionnaires	Only fully completed questionnaires were analyzed. Since all items were mandatory, no missing data existed.
Analysis - Atypical timestamps	Extremely short response times were considered invalid, and such responses were excluded.
Analysis - Statistical correction	No weighting or statistical correction was applied. The study aimed to explore factors associated with attitudes toward data sharing rather than producing nationally representative prevalence estimates.
