# Supplementary material for: Mobile Health App Acceptance in Japan’s Aging Society: Multigroup Structural Equation Modeling Based on the Extended Unified Theory of Acceptance and Use of Technology and eHealth Literacy Frameworks
Source: JMIR Mhealth Uhealth. 2026 Jun 9;14:e87832. doi: 10.2196/87832 (PMC13291735; doi:10.2196/87832)
Supplement: Multimedia Appendix 2 [file mhealth_v14i1e87832_app2.docx]

Table S1 Construct items and Mean SD

| Construct | Item | Mean | SD | Source |
| --- | --- | --- | --- | --- |
| Performance Expectancy (PE) | Medical and healthcare services utilizing mobile technology can improve overall well-being | 3.16 | 0.76 | Venkatesh et al. (2012);  Cho (2016); Hoque & Sorwar (2017) |
|  | Medical and healthcare services utilizing mobile technology can be used to manage my health, prevent disease, and reduce stress | 3.21 | 0.76 |  |
|  | Medical and healthcare services utilizing mobile technology can help me improve my health and medical care efficiency | 3.33 | 0.73 |  |
| Effort Expectancy (EE) | The operation of medical and healthcare services utilizing mobile technology is simple and easy to understand | 3.06 | 0.76 | Venkatesh et al. (2012);  Zhang et al. (2014) |
|  | Medical and healthcare services that utilize mobile technology can be easily incorporated into daily life | 3.16 | 0.8 |  |
| Social Influence (SI) | My family doctor will recommend medical and healthcare services that utilize mobile technology | 2.94 | 0.85 | Venkatesh et al. (2012);  Alam et al. (2020);  Cao et al. (2022) |
|  | My family will recommend medical and healthcare services that utilize mobile technology | 2.89 | 0.79 |  |
|  | My friends will recommend medical and healthcare services that utilize mobile technology | 2.87 | 0.83 |  |
| Facilitating Conditions (FC) | I can set up the technical environment necessary to use mobile health | 2.94 | 0.86 | Venkatesh et al. (2012); Hoque & Sorwar (2017); Alam et al. (2020) |
|  | I have the technical knowledge necessary to use mobile health | 2.8 | 0.92 |  |
|  | I can get tech support if I experience technical issues when using mobile health | 3.01 | 0.9 |  |
| Perceived risk | I feel apprehensive about using mobile health technology | 2.59 | 0.92 | Pavlou (2003); Featherman & Pavlou (2003); Anderson & Agarwal (2011) |
|  | I hesitate to use the mobile health technology to make an irreparable mistake | 3.03 | 0.99 |  |
|  | I have concerns about the use of mobile health technology | 2.79 | 1.02 |  |
| Self-Efficacy (SE) | I feel like I'm in control of my life | 2.85 | 0.92 | Bandura (1977); Compeau & Higgins (1995); Dou et al. (2017) |
|  | Despite the difficulties, I managed to cope with everyday life | 3.32 | 0.8 |  |
|  | I am confident that I will be able to overcome the difficulties | 3.11 | 0.83 |  |
| Distrust (DT) | The diagnostic results provided by mobile health technologies lack reliability | 3.09 | 0.78 | McKnight et al. (2002); Lankton et al. (2015); Mou & Cohen (2023); Nebeker et al. (2023) |
|  | There are concerns that mobile health services may encourage self-diagnosis without professional consultation | 3.16 | 0.74 |  |
|  | Mobile health technologies are sometimes inaccurate | 3.45 | 0.8 |  |
| Behavioral Intention (BI) | I would like to try using mobile devices for health management and medical services | 3.03 | 0.93 | Venkatesh et al. (2012); Terhorst et al. (2021); Yang et al. (2024) |
|  | If I have the opportunity, I would like to use mobile digital technology for health and medical care | 3.12 | 0.91 |  |
|  | I intend to use medical and healthcare services utilizing mobile technology on a regular basis | 2.92 | 0.87 |  |
| eHealth Literacy (eHL) | I know how to find helpful health resources on the Internet | 2.85 | 1.002 | Norman & Skinner (2006); Neter & Brainin (2012); Mitsutake et al. (2012); Paige et al. (2017); Hsu et al. (2023) |
|  | I know how to use the Internet to answer my health questions | 2.85 | 0.968 |  |
|  | I know what health resources are available on the Internet | 3 | 0.988 |  |
|  | I know where to find helpful health resources on the Internet | 2.99 | 0.953 |  |
|  | I know how to use the health information I find on the Internet to help me | 2.96 | 0.961 |  |
|  | I have the skills I need to evaluate the health resources I find on the Internet | 2.7 | 0.935 |  |
|  | I can tell high quality health resources from low quality health resources on the Internet | 2.7 | 0.939 |  |
|  | I feel confident in using information from the Internet to make health decisions | 2.78 | 0.916 |  |

*Note.*  5-point Likert scale: 1 = Strongly Disagree, 2 = Disagree, 3 = Neither Agree nor Disagree, 4 = Agree, 5 = Strongly Agree. All items were administered in Japanese; English translations provided here for international readership.
