# Supplementary material for: Mobile Health App Acceptance in Japan’s Aging Society: Multigroup Structural Equation Modeling Based on the Extended Unified Theory of Acceptance and Use of Technology and eHealth Literacy Frameworks
Source: JMIR Mhealth Uhealth. 2026 Jun 9;14:e87832. doi: 10.2196/87832 (PMC13291735; doi:10.2196/87832)
Supplement: Multimedia Appendix 3 [file mhealth_v14i1e87832_app3.docx]

Supplementary Table S2. Descriptive statistics and correlations

| Variable | M | SD | 1 | 2 | 3 | 4 | 5 | 6 | 7 | 8 | 9 |
| --- | --- | --- | --- | --- | --- | --- | --- | --- | --- | --- | --- |
| 1. SE | 3.09 | 0.69 | - | 0.46 | 0.4 | -0.07 | 0.41 | 0.46 | 0.44 | 0.01 | 0.36 |
| 2. SI | 2.9 | 0.73 | 0.46 | - | 0.44 | 0.07 | 0.63 | 0.73 | 0.7 | -0.08 | 0.65 |
| 3. eL | 2.86 | 0.79 | 0.4 | 0.44 | - | -0.13 | 0.45 | 0.56 | 0.61 | 0.07 | 0.49 |
| 4. PR | 2.8 | 0.84 | -0.07 | 0.07 | -0.13 | - | -0.08 | -0.13 | -0.15 | 0.19 | -0.14 |
| 5. PE | 3.23 | 0.67 | 0.41 | 0.63 | 0.45 | -0.08 | - | 0.76 | 0.56 | 0.01 | 0.68 |
| 6. EE | 3.11 | 0.71 | 0.46 | 0.73 | 0.56 | -0.13 | 0.76 | - | 0.76 | 0.05 | 0.74 |
| 7. FC | 2.92 | 0.77 | 0.44 | 0.7 | 0.61 | -0.15 | 0.56 | 0.76 | - | 0.06 | 0.67 |
| 8. DT | 3.23 | 0.63 | 0.01 | -0.08 | 0.07 | 0.19 | 0.01 | 0.05 | 0.06 | - | -0.03 |
| 9. BI | 3.02 | 0.83 | 0.36 | 0.65 | 0.49 | -0.14 | 0.68 | 0.74 | 0.67 | -0.03 | - |

*Note.* M = Mean; SD = Standard Deviation;. SE = Self-Efficacy; SI = Social Influence; eL = e-Health Literacy; PR = Privacy Risk; PE = Performance Expectancy; EE = Effort Expectancy; FC = Facilitating Conditions; DT = Device Type; BI = Behavioral Intention. All correlations significant at p < .001 except: SE-PR, SE-DT, SI-PR, SI-DT, eL-DT, PE-DT, EE-DT, FC-DT, DT-BI (p > .05); PR-PE (p < .05).
