# Supplementary figures and images for: Mobile Health App Acceptance in Japan’s Aging Society: Multigroup Structural Equation Modeling Based on the Extended Unified Theory of Acceptance and Use of Technology and eHealth Literacy Frameworks
Source: JMIR Mhealth Uhealth. 2026 Jun 9;14:e87832. doi: 10.2196/87832 (PMC13291735; doi:10.2196/87832)

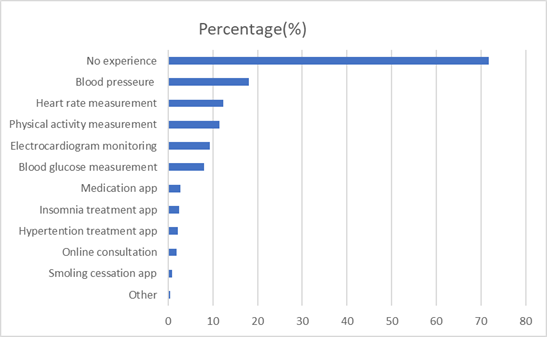

Supplement: Multimedia Appendix 4 [file mhealth_v14i1e87832_app4.png]
