# Supplementary material for: Mobile Health App Acceptance in Japan’s Aging Society: Multigroup Structural Equation Modeling Based on the Extended Unified Theory of Acceptance and Use of Technology and eHealth Literacy Frameworks
Source: JMIR Mhealth Uhealth. 2026 Jun 9;14:e87832. doi: 10.2196/87832 (PMC13291735; doi:10.2196/87832)
Supplement: Multimedia Appendix 5 [file mhealth_v14i1e87832_app5.docx]

Supplementary Table 3

Confirmatory Factor Analysis Results: Factor Loadings, Reliability, and Validity Indices for Constructs and Items

| **Construct** | **Item** | **Unstandardized Loading** | **t-value** | **Standardized Loading** | **AVE** | **CR** | **Cronbach's α** | **MSV** |
| --- | --- | --- | --- | --- | --- | --- | --- | --- |
| SE | se1 | 1 | — | 0.823 | 0.501 | 0.752 | 0.731 | 0.214 |
|  | se2 | 0.78 | 12.605*** | 0.675 |  |  |  |  |
|  | se3 | 0.874 | 12.357*** | 0.656 |  |  |  |  |
| SI | si1 | 1 | — | 0.803 | 0.644 | 0.844 | 0.871 | 0.54 |
|  | si2 | 0.927 | 20.597*** | 0.767 |  |  |  |  |
|  | si3 | 1.045 | 19.107*** | 0.824 |  |  |  |  |
| PR | pr1 | 1 | — | 0.921 | 0.658 | 0.852 | 0.83 | 0.035 |
|  | pr2 | 0.853 | 17.165*** | 0.796 |  |  |  |  |
|  | pr3 | 0.673 | 14.561*** | 0.659 |  |  |  |  |
| PE | pe1 | 1 | — | 0.853 | 0.687 | 0.868 | 0.868 | 0.578 |
|  | pe2 | 0.984 | 20.240*** | 0.86 |  |  |  |  |
|  | pe3 | 0.866 | 19.118*** | 0.788 |  |  |  |  |
| EE | ee1 | 1 | — | 0.799 | 0.659 | 0.795 | 0.779 | 0.578 |
|  | ee2 | 1.084 | 18.640*** | 0.825 |  |  |  |  |
| DT | dt1 | 1 | — | 0.773 | 0.5 | 0.749 | 0.739 | 0.035 |
|  | dt2 | 0.908 | 11.337*** | 0.731 |  |  |  |  |
|  | dt3 | 0.792 | 10.698*** | 0.612 |  |  |  |  |
| FC | fc1 | 1 | — | 0.848 | 0.65 | 0.847 | 0.827 | 0.578 |
|  | fc2 | 1.059 | 21.009*** | 0.846 |  |  |  |  |
|  | fc3 | 0.839 | 16.291*** | 0.693 |  |  |  |  |
| BI | bi1 | 1 | — | 0.841 | 0.761 | 0.905 | 0.907 | 0.543 |
|  | bi2 | 0.989 | 30.942*** | 0.869 |  |  |  |  |
|  | bi3 | 1.023 | 22.853*** | 0.901 |  |  |  |  |
| eHL | ehl1 | 1 | — | 0.789 | 0.665 | 0.941 | 0.935 | 0.367 |
|  | ehl2 | 0.981 | 21.021*** | 0.751 |  |  |  |  |
|  | ehl3 | 0.971 | 20.338*** | 0.745 |  |  |  |  |
|  | ehl4 | 1.122 | 19.760*** | 0.847 |  |  |  |  |
|  | ehl5 | 1.143 | 19.999*** | 0.856 |  |  |  |  |
|  | ehl6 | 1.185 | 20.549*** | 0.872 |  |  |  |  |
|  | ehl7 | 1.117 | 20.006*** | 0.836 |  |  |  |  |
|  | ehl8 | 1.081 | 18.442*** | 0.785 |  |  |  |  |

Note. N = 960. AVE = Average Variance Extracted; CR = Composite Reliability; MSV = Maximum Shared Variance. ***P < .001. All constructs demonstrated adequate reliability (CR ≥ .70, Cronbach's α ≥ .73). Convergent validity was supported for most constructs (AVE ≥ .50), with SE (AVE = .496) and DT (AVE = .489) showing marginally acceptable values. Discriminant validity was confirmed using the Fornell–Larcker criterion (AVE > MSV) for all constructs. SE = Self-Efficacy; SI = Social Influence; PR = Perceived Risk; PE = Performance Expectancy; EE = Effort Expectancy; DT = Distrust; FC = Facilitating Conditions; BI = Behavioral Intention; eHL = eHealth Literacy.
